# Patterns of IgA Autoantibody Generation, Inflammatory Responses and Extracellular Matrix Metabolism in Patients with Alcohol Use Disorder

**DOI:** 10.3390/ijms241713124

**Published:** 2023-08-23

**Authors:** Onni Niemelä, Aini Bloigu, Risto Bloigu, Ulla Nivukoski, Johanna Kultti, Heidi Pohjasniemi

**Affiliations:** 1 Department of Laboratory Medicine and Medical Research Unit, Seinäjoki Central Hospital, 60220 Seinäjoki, Finland; ulla.nivukoski@hyvaep.fi (U.N.); johanna.kultti@hyvaep.fi (J.K.); heidi.pohjasniemi@hyvaep.fi (H.P.); 2 Faculty of Medicine and Health Technology, Tampere University, 33014 Tampere, Finland; 3 Research Unit of Population Health, Faculty of Medicine, University of Oulu, 90220 Oulu, Finland; abloigu@outlook.com; 4 Infrastructure of Population Studies, Faculty of Medicine, University of Oulu, 90220 Oulu, Finland; rbloigu@gmail.com

**Keywords:** alcoholic liver disease, autoantibodies, biomarker, fibrosis, inflammation, interleukin, protein desialylation

## Abstract

Recent data have emphasized the role of inflammation and intestinal immunoglobulin A (IgA) responses in the pathogenesis of alcoholic liver disease (ALD). In order to further explore such associations, we compared IgA titers against antigens targeted to ethanol metabolites and tissue transglutaminase with pro- and anti-inflammatory mediators of inflammation, markers of liver status, transferrin protein desialylation and extracellular matrix metabolism in alcohol-dependent patients with or without liver disease and in healthy controls. Serum IgAs against protein adducts with acetaldehyde (HbAch-IgA), the first metabolite of ethanol, and tissue transglutaminase (tTG-IgA), desialylated transferrin (CDT), pro- and anti-inflammatory cytokines, markers of liver status (GT, ALP) and extracellular matrix metabolism (PIIINP, PINP, hyaluronic acid, ICTP and CTx) were measured in alcohol-dependent patients with (*n* = 83) or without (*n* = 105) liver disease and 88 healthy controls representing either moderate drinkers or abstainers. In ALD patients, both tTG-IgA and HbAch-IgA titers were significantly higher than those in the alcoholics without liver disease (*p* < 0.0005 for tTG-IgA, *p* = 0.006 for Hb-Ach-IgA) or in healthy controls (*p* < 0.0005 for both comparisons). The HbAch-IgA levels in the alcoholics without liver disease also exceeded those found in healthy controls (*p* = 0.0008). In ROC analyses, anti-tTG-antibodies showed an excellent discriminative value in differentiating between ALD patients and healthy controls (AUC = 0.95, *p* < 0.0005). Significant correlations emerged between tTG-IgAs and HbAch-IgAs (r_s_ = 0.462, *p* < 0.0005), CDT (r_s_ = 0.413, *p* < 0.0001), GT (r_s_ = 0.487, *p* < 0.0001), alkaline phosphatase (r_s_ = 0.466, *p* < 0.0001), serum markers of fibrogenesis: PIIINP (r_s_ = 0.634, *p* < 0.0001), hyaluronic acid (r_s_ = 0.575, *p* < 0.0001), ICTP (r_s_ = 0.482, *p* < 0.0001), pro-inflammatory cytokines IL-6 (r_s_ = 0.581, *p* < 0.0001), IL-8 (r_s_ = 0.535, *p* < 0.0001) and TNF-α (r_s_ = 0.591, *p* < 0.0001), whereas significant inverse correlations were observed with serum TGF-β (r_s_ = −0.366, *p* < 0.0001) and CTx, a marker of collagen degradation (r_s_ = −0.495, *p* < 0.0001). The data indicate that the induction of IgA immune responses toward ethanol metabolites and tissue transglutaminaseis a characteristic feature of patients with AUD and coincides with the activation of inflammation, extracellular matrix remodeling and the generation of aberrantly glycosylated proteins. These processes appear to work in concert in the sequence of events leading from heavy drinking to ALD.

## 1. Introduction

Accumulating data have indicated that heavy alcohol intake triggers inflammation, aberrant immune responses and excessive deposition of the extracellular matrix (ECM) in the liver [1,2,3,4,5,6,7,8]. Among the most typical manifestations of humoral immune responses in patients with alcohol use disorders (AUDs) are elevated serum IgA immunoglobulin levels and tissue deposition of IgAs in the liver and kidney [9,10,11,12]. The cellular and molecular mechanisms driving such responses have, however, remained unclear.

Earlier studies have demonstrated that alcohol metabolism leads to the formation of reactive toxic metabolites, which may create functional and structural changes in proteins and cellular constituents [13,14,15,16]. Protein modifications with acetaldehyde, the first metabolite of ethanol, metabolic products of oxidative stress and lipopolysaccharide (LPS) can also induce immune responses against the corresponding neoantigens [12,15,16,17,18,19,20]. Such humoral immune responses to ethanol metabolites primarily consist of IgA isotype antibodies [12,14,20]. Excessive ethanol consumption can also lead to the mounting of IgA antibodies to tissue transglutaminase (tTG) [21], which is also known as a highly specific autoantigen of celiac disease [22,23,24,25], although such antibodies have also been reported in neurodegenerative conditions [26,27] and in diabetes [28]. Tissue transglutaminase is a multifunctional enzyme that catalyzes the crosslinking of proteins by epsilon-(gamma-glutamyl) lysine isopeptide bonds. The status of tTG expression also seems to be important for IgA tissue binding [29,30]. Recent studies in patients with IgA nephropathy have suggested that the generation of disease-specific IgAs may also be associated with aberrant protein glycosylation and sialic acid deficiencies in proteins [31], which is also known as a distinguishing characteristic of patients with chronic alcohol abuse [32].

In AUD patients, both the amount of drinking and the severity of tissue injury seem to influence the status of inflammation [4,5,33,34,35,36,37,38,39]. Heavy alcohol drinking and chronic inflammation also stimulate the deposition of ECM proteins and liver fibrosis characterized by alterations in both the amount and composition of the ECM [2,40,41,42,43,44]. Currently, however, only limited information is available on comparisons of the distinct IgA antibody responses, the status of inflammation and ECM metabolism in individuals with a wide range of alcohol intake and associated liver damage. In this study, we examined the generation of IgA autoantibodies against ethanol metabolites and tissue transglutaminase, pro- and anti-inflammatory mediators of inflammation and markers of connective tissue metabolism in alcoholic patients with or without liver disease. The biomarker levels were also compared with the data obtained from markers of protein desialylation and liver status. Our findings indicate distinct interactions between alcohol-induced humoral immune responses and the inflammatory and fibrogenic pathways of tissue damage in patients with AUD.

## 2. Results

Table 1 summarizes the main demographic characteristics of the study population, which comprised 188 patients with AUD and 88 healthy controls representing either moderate drinkers or abstainers. Among the AUD patients, there were 83 patients who had been diagnosed with alcoholic liver disease (ALD) and 105 individuals who were alcohol-dependent and had been admitted for detoxification but were devoid of any apparent clinical and laboratory signs of significant liver disease.

**Figure 1:**
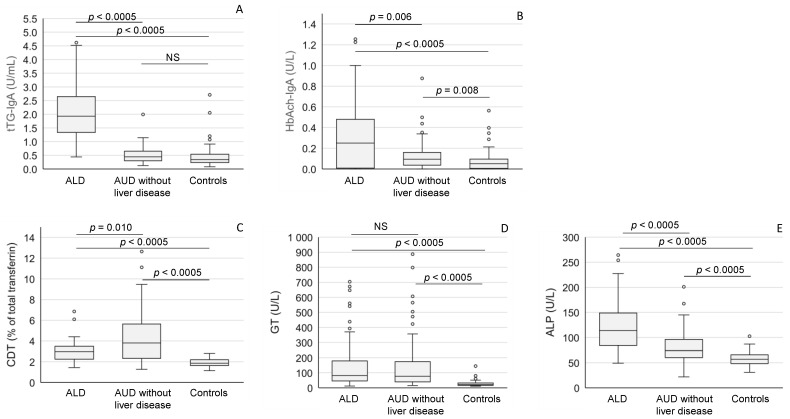
Anti-tTG IgA antibodies (**A**), anti-acetaldehyde-adduct IgA (HbAch-IgA) antibodies (**B**), desialylated transferrin (CDT) (**C**), gamma-glutamyl transferase (GT) (**D**) and alkaline phosphatase (ALP) (**E**) levels in patients with alcoholic liver disease (ALD), and alcohol use disorder (AUD) without liver disease and healthy controls. The data are expressed as medians and interquartile ranges. NS = not significant.

The titers of tTG-IgA antibodies in ALD patients (median 1.93 U/L [IQR 1.34–2.65 U/L]) were significantly higher than those in the healthy controls (median 0.34 U/L [IQR 0.24–0.54 U/L], *p* < 0.0005) or in the alcoholics without liver disease (median 0.45 U/L [IQR 0.30–0.65 U/L], *p* < 0.0005) (Figure 1A). The highest titers of IgAs against hemoglobin-acetaldehyde (Hb-Ach) adducts were also observed in the alcoholics with liver disease (median 0.25 U/L [IQR 0.01–0.48 U/L]), being significantly higher than those in the alcoholics without liver disease (median 0.09 U/L [IQR 0.04–0.16 U/L], *p* = 0.006) or in the healthy controls (median 0.05 U/L [IQR 0.01–0.10 U/L], *p* = 0.0005) (Figure 1B). HbAch-IgA titers in the heavy drinkers without liver disease also exceeded the levels found in the healthy controls (*p* = 0.008) (Figure 1B). The values of desialylated (carbohydrate-deficient) transferrin (CDT) were highest in the group of heavy drinkers without liver disease (Figure 1C). Serum gamma-glutamyl transferase (GT) activities were high in both AUD subgroups (Figure 1D), whereas alkaline phosphatase (ALP) activities were most strikingly elevated in ALD patients (Figure 1E).

**Figure 2:**
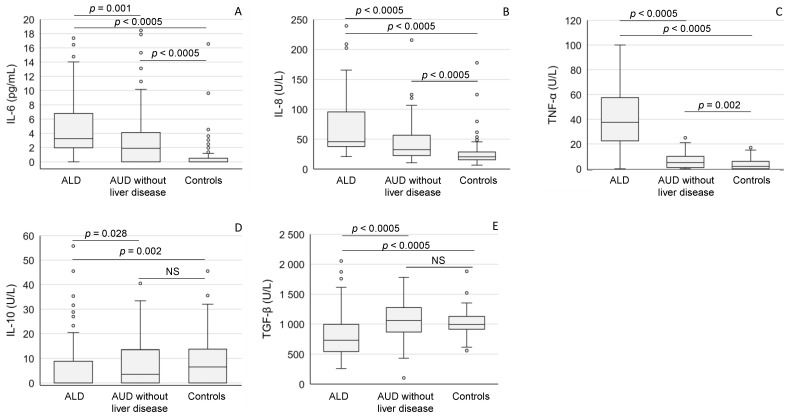
The mediators of inflammation (**A**: IL-6; **B**: IL-8; **C**: TNF-α; **D**: IL-10; **E**: TGF-β) in patients with alcoholic liver disease (ALD), and AUD without liver disease and in healthy controls. The data are expressed as medians and interquartile ranges. For abbreviations, see Table 2. NS = not significant.

ALD patients showed markedly higher values of pro-inflammatory mediators (IL-6, IL-8 and TNF-α) when compared to patients without liver disease or healthy controls (Figure 2A−C). The levels of these cytokines in the heavy drinkers without liver disease also significantly exceeded those found in healthy controls (Figure 2A−C), whereas the levels of anti-inflammatory IL-10 cytokine and TGF-β were relatively low in ALD patients when compared to the other study subgroups (Figure 2D,E).

**Figure 3:**
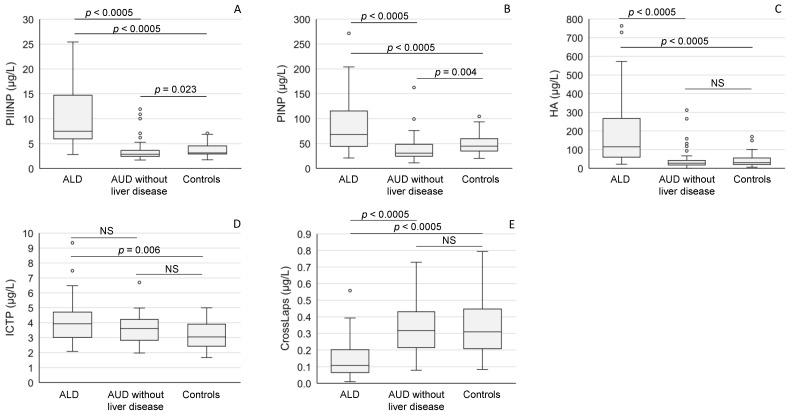
The concentrations of serum markers of connective tissue metabolism ((**A**): PIIINP; (**B**): PINP; (**C**): HA; (**D**): ICTP, (**E**): CrossLaps) in patients with alcoholic liver disease (ALD), and AUD without liver disease and healthy controls. The data are expressed as medians and interquartile ranges. For abbreviations, see Table 2. NS = not significant.

ALD status was also a major determinant in the balance of serum biomarkers reflecting extracellular matrix synthesis and degradation. Serum aminoterminal propeptide of type III procollagen (PIIINP) (Figure 3A), aminoterminal propeptide of type I procollagen (PINP) (Figure 3B), hyaluronic acid (HA) (Figure 3C) and carboxyterminal telopeptide of type I procollagen (ICTP) (Figure 3D) levels were all high among ALD patients. In contrast, the concentrations of serum CTx (CrossLaps), a marker of collagen degradation, were low in ALD patients when compared to the values in the alcoholics without liver disease or healthy controls (*p* < 0.0005 for both comparisons) (Figure 3E).

The diagnostic ability of the various biomarkers in distinguishing between the subgroups of patients with AUD and the control group is summarized in Table 2. The sensitivity and specificity of serum tTG-IgA antibodies (AUC 0.95, confidence interval 0.91–0.99) and TNF-α (AUC 0.97, confidence interval 0.94–1.00) in correctly classifying the ALD patients were notably higher than those of the other biomarkers in these comparisons. For most biomarkers, ALD status was the main determinant for abnormalities, whereas HbAch-IgA, desialylated transferrin (CDT) and GT appeared to be more sensitive to alcohol consumption per se (Table 2).

Table 3 summarizes the correlations between the various study parameters. The levels of tTG-IgAs correlated significantly with HbAch-IgAs (r_s_ = 0.462, *p* < 0.0005), CDT (r_s_ = 0.413, *p* < 0.0001), GT (r_s_ = 0.487, *p* < 0.0001), alkaline phosphatase (r_s_ = 0.466, *p* < 0.0001), serum markers of fibrogenesis: PIIINP (r_s_ = 0.634, *p* < 0.0001), hyaluronic acid (r_s_ = 0.575, *p* < 0.0001), ICTP (r_s_ = 0.482, *p* < 0.0001), cytokines IL-6 (r_s_ = 0.581, *p* < 0.0001), IL-8 (r_s_ = 0.535, *p* < 0.0001), TNF-α (r_s_ = 0.591, *p* < 0.0001) and inversely with serum TGF-β (r_s_ = −0.366, *p* < 0.0001) and CTx, a biomarker of collagen degradation (r_s_ = −0.495, *p* < 0.0001). The correlation between these antibodies was also significant in the subgroups consisting of moderate drinkers and abstainers (r_s_ = 0.598, *p* < 0.0001) or heavy drinkers without liver disease (r_s_ = 0.503, *p* < 0.0001) only. In the latter subgroup, tTG-IgA levels also showed significant correlations with desialylated transferrin (r_s_ = 0.430, *p* < 0.01), IL-6 (r_s_ = 0.589, *p* < 0.01) and TNF-α (r_s_ = 0.534, *p* < 0.01).

## 3. Discussion

Our study comparing IgA autoantibody responses to ethanol metabolites and tissue transglutaminase, mediators of inflammation, the status of protein desialylation and the metabolism of the extracellular matrix in AUD patients with various stages of liver disease severity reveals distinct coinciding phenomena in these metabolic pathways. The findings also suggest an important role of gut-derived immune responses as drivers of inflammation and fibrogenesis in AUD.

Excessive ethanol consumption can modify intestinal immunity through a variety of mechanisms including changes in microbial flora, damage to epithelial cells and compromised T-cell and neutrophil function. These may lead to alterations in intestinal permeability and gut barrier function with the leakage of micro-organisms into the circulation, which are currently recognized as crucial determinants in the interactions between diet and various disease outcomes [45,46,47,48]. In alcohol consumers, intestinally induced immune responses in B-cells of ethanol-exposed epithelial tissues may also be associated with various extra-intestinal disease manifestations, including problems in the liver, kidney or brain [9,14,15,18,20,48,49].

IgA isotype antibodies, which are generated daily in abundant amounts from antibody-secreting cells in the mucosal lumen, arethe main immune effectors in the gastrointestinal tract capable of recruiting neutrophils, monocytes and macrophages, thereby playing a pivotal role in immune defense against orally ingested antigens [31,50,51]. The GI tract is rich in enzymes metabolizing ethanol to acetaldehyde, which has the ability to generate stable adducts with proteins, interfere with cellular functions and increase the risk of gastrointestinal tract carcinogenesis in heavy alcohol drinkers and in individuals with a genetic predisposition to high acetaldehyde levels [14,52,53,54]. Adduct formation can also induce IgA antibodies, which have been previously observed in a high percentage of patients with AUD [11,12,14,55,56]. Such immune responses may be required for the exclusion and neutralization of the neoantigens although an overwhelming antigenic stimulation could also cause a release of inflammatory mediators and aggravate tissue damage [14,57,58].

Heavy alcohol drinking was also found to induce IgAs to tTG, which has been previously established as a specific target antigen in celiac disease [22,23,24,25]. These antibodies were most strikingly elevated in ALD patients suggesting a connection between liver disease status and the perpetuation of the immune responses [21,59,60,61,62,63]. However, elevated titers also occurred in alcohol consumers without liver disease coinciding with increased levels of antibodies against the ethanol-derived epitopes indicating that heavy alcohol drinking per se is associated with autoimmune responses against the gut-derived antigens. Upon alcohol-induced mucosal damage, a stronger antigenic presentation and breakdown of immune tolerance may take place. It remains to be established to what extent such phenomena could also contribute to commonly found gastrointestinal symptoms following heavy alcohol intake and adverse effects in the liver, which is the primary site of ethanol metabolism. Interestingly, the present data also show significant correlations between anti-tTG antibodies and desialylated transferrin even in alcohol consumers without liver disease. A lack of sialic acid residues in transferrin is known as a highly specific feature of chronic heavy drinking [32]. On the other hand, studies in patients with IgA nephropathy, which is also a possible unfavorable outcome of heavy alcohol intake [9], have indicated that aberrant glycosylation and sialic acid deficiency in proteins is a typical characteristic of the neoantigens capable of inducing IgA responses and nephritogenic immune complexes [31]. It should further be noted that the cellular expression of tTG appears to be important for IgA tissue-binding properties [29].

The most important biological function of tTG is the formation of epsilon (gamma-glutamyl) lysine isopeptide linkages, which yield protective, preventive and extracellular matrix remodeling effects in tissue repair processes [30,64,65]. In patients with celiac disease, tTG has been shown to form high-molecular-weight complexes with gliadin and novel cross-linking patterns with ECM proteins thereby stimulating intestinal inflammation and associated autoimmune phenomena [66,67,68]. In an analogous manner, high prevailing concentrations of acetaldehyde could accelerate protein cross-linking under appropriate reducing conditions [14,15,49,56,69]. It is possible that the above neoantigens could also have similar conformational and sequential epitopes leading to immunological cross-reactivity and sensitized mucosal immunity in response to heavy alcohol drinking. The onset of ALD may lead to additional sources of antigenic stimulation, aberrant tTG activation and additive effects in alcohol-exposed tissues or in conditions where alcohol consumption coexists with other morbidities such as neurodegenerative conditions or diabetes [26,27,28]. It should further be noted that the amounts of circulating tTG antibodies have previously also been linked with increased all-cause mortality [70,71].

Based on the above considerations, alcohol-induced changes in the gut may play a significant role in the regulation of inflammation and the development of liver disease in AUD patients [5,48,72,73,74,75,76,77,78]. The finding that IgA antibody responses in alcohol users devoid of liver disease were also found to correlate with pro-inflammatory cytokines suggests that such inflammatory responses also characterize the early phase of the sequence of events leading from heavy drinking to liver disease. With disease progression, an altered balance of pro- and anti-inflammatory cytokines favoring pro-inflammatory mediators can be found together with high neutrophil counts, active inflammation and increased activities of liver-derived enzymes in the circulation [6,36,37,79,80,81,82]. This switch in the cytokine status could also facilitate hepatic fibrogenesis under conditions of overwhelming ethanol-derived antigen presentation, generation of endotoxin and gut bacterial products, oxidative stress and deficient anti-inflammatory capacity [14,17,81,83,84,85,86,87]. Interestingly, ALP, which has the ability to detoxify endotoxin [88], was also found to show a correlation with the immune responses and the mediators of inflammation.

Previously, abnormalities in several types of individual inflammatory mediators have been reported in patients with ALD [5,37,89,90,91,92]. Studies have indicated that pro-inflammatory cytokine levels correlate with disease severity and prognosis [37,92,93,94,95]. Here, strong correlations were found to emerge between anti-tTG IgAs and IL-6, IL-8 and TNF-α, which are key mediators of inflammation and oxidative stress and associateare d with the upregulation of ECM proteins and long-term survival in alcoholics [87,92,96,97,98,99,100,101,102]. TNF-α has been shown to be closely linked with the status of inflammation and necrosis due to its ability to attract neutrophils and regulate macrophage production [87,103,104,105,106]. Interleukin-8 is also a neutrophil chemoattractant, and its activated expression leads to fibrogenesis and decreased hepatocyte survival [107]. IL-6 can act in either a pro-inflammatory or anti-inflammatory manner, the increased values signaling a need for liver regeneration and resistance to injury [5,103,108,109,110]. IL-10 is a cytokine with potent anti-inflammatory properties acting to suppress inflammatory processes and inhibit TNF-α [87]. The guidance of the immune system toward the pro-inflammatory direction favoring cell injury may be determined by several factors, including the prevailing status of the metabolic burden and intracellular pH [111]. Nevertheless, based on current data, it appears that in ALD patients, the anti-inflammatory capacity is not sufficient to keep pace with the pro-inflammatory stimuli.

In support of immunological mechanisms contributing to the progression of ALD, clinical observations indicate that ALD patients often show deterioration for long periods after hospitalization and cessation of ethanol consumption [57,112]. An excess release of pro-inflammatory mediators can maintain over-activation of the sympathetic nervous system, oxidative stress and an immunocompromised status [83,113,114,115]. Our recent follow-up studies have indicated that key mediators of inflammation stay elevated in most heavy drinkers during a period of 1−2 weeks of abstinence [6]. Some of the immune abnormalities following alcohol drinking may also be dependent on the actual quantities of ethanol consumed [39].

The present data further demonstrate highly significant correlations between the anti-tTG-IgA autoantibodies and markers of connective tissue metabolism indicating that the immune responses also parallel the stimulation of ECM metabolism. The ECM synthesis markers were strongly associated with pro-inflammatory cytokines, whereas TGF-β, which has pleiotropic effects in the developmentof fibrosis in alcoholics [116,117], showed a negative association. The development of liver fibrosis in AUD patients constitutes a major determinant of prognosis and is characterized by significant alterations in both the amount and composition of the ECM [32,40,41,44,118,119]. ECM protein secretion can be stimulated by the activation of hepatic stellate cells as a result of inflammation and the generation of toxic metabolic products of ethanol [40,120,121,122,123]. Tissue transglutaminase also seems to have a role in regulating the organization of the ECM [65]. In accordance with a disturbed ECM turnover, current biomarker data indicate deviations in the balance between collagen synthesis and degradation in ALD patients. The concentrations of a collagen degradation marker (CTx) were found to follow different kinetics when compared to markers of interstitial collagens or hyaluronic acid. The failure of collagen removal to keep pace with excessive synthesis in the pathogenesis of liver fibrosis has been previously supported by findings indicating decreased collagenase and matrix metalloproteinase activities and overexpression of tissue inhibitors of matrix metalloproteinases (TIMP) in patients with severe liver disease [119,124,125]. Combinations of biomarkers reflecting ECM synthesis and degradation have also been recently utilized in the development of more sensitive approaches for assessing fibrogenesis by using specifically designed algorithms for selected ECM components, such as PIIINP, hyaluronic acid and TIMP [126].

Some limitations of the study should be pointed out. Due to ethical considerations, the group of heavy drinkers admitted for detoxification were not biopsied. Therefore, although these individuals were devoid of previous medical records or current clinical and laboratory signs of liver disease, we cannot rule out the possibility of mild to moderate liver disease, such as fatty change, in these individuals. Structured data on the use of over-the-counter medication, which could aggravate liver disease in an unexpected manner, were not available. Because of the relatively low number of participants, separate statistical analyses for men and women were not carried out. Due to well-known differences in the physiology of alcohol metabolism and susceptibilities to tissue damage between men and women, this issue clearly warrants further studies with larger samples. It should also be noted that in this work, abstainers and moderate drinkers were pooled because they both represented apparently healthy individuals without any signs of alcohol use disorder. This approach also allowed larger sample sizes for the statistical comparisons. Separate comparisons of these subgroups indicated, however, no significant differences in the current study parameters.

Taken together, the present data demonstrate distinct IgA isotype immune responses against tissue transglutaminase and antigens induced by ethanol metabolites in heavy alcohol drinkers. These changes also coincide with changes in mediators of inflammation, desialylation of transferrin and ECM metabolism suggesting a key role of the immune and inflammatory mechanism in AUD. In light of recent findings indicating that elevated anti-tTG antibodies may also be associated with higher all-cause mortality risk and the high diagnostic accuracy of such antibodies in differentiating patients with ALD from AUD without apparent liver disease, future studies appear warranted to examine the predictive and prognostic value of such immune responses in alcoholic patients at risk for developing liver cirrhosis. These findings should also be implicated in further studies on the primary mechanisms of alcohol-induced tissue damage.

## 4. Materials and Methods

### 4.1. Participants

The present study population included 188 patients presenting with DSM-5 criteria of alcohol use disorder (AUD) and a well-documented clinical history of chronic heavy alcohol drinking. The patients were characterized by severe alcohol dependence, marked alcohol tolerance and a strong urge to drink increasing amounts of alcohol to reach the desired rewarding effects. They had also shown withdrawal symptoms upon cessation of drinking and had been unable to stop alcohol consumption despite obvious medical, occupational and social problems.

Among the patients with AUD, there were 83 patients with clinical, laboratory and morphological evidence of alcoholic liver disease (ALD) (Table 1). The liver histology in these individuals ranged from mild fibrosis and fatty change to cirrhosis with a wide range of morphological abnormalities related to alcoholic hepatitis. In addition, we studied 105 heavy alcohol drinkers who had been hospitalized for detoxification but did not show obvious clinical and laboratory signs of liver disease despite a history of continuous ethanol consumption or binge drinking, which had consisted of a mean total of 950 g of ethanol (corresponding to approximately 10 standard drinks per day) over the period of one month preceding blood sampling, as assessed by detailed personal interviews using a timeline followback technique. Due to ethical considerations, these patients were not biopsied. Controls were 88 healthy volunteers, who were either abstainers or mild to moderate drinkers. Moderate drinkers were defined as individuals who were able to limit their alcohol intake to two drinks or less in a day for men or one drink or less in a day for women on days when alcohol was consumed. None of these individuals had any previous history of alcohol abuse.

The study subjects were devoid of clinical and laboratory records of significant comorbid substance abuse, active infections or clinical signs of active extrahepatic inflammation. The mean duration of abstinence prior to the sampling was 2 ± 1 days.

All subjects gave their informed consent for the study. The protocol was approved by the local ethical committee of The Hospital District of South Ostrobothnia and Tampere University, and the study was conducted according to the provisions of the Declaration of Helsinki.

### 4.2. Laboratory Methods

Serum samples were prepared by centrifugation (1500 × *g* for 10 min) and stored at −70 °C until analysis. Blood chemistry analyses were carried out using standard clinical chemical methods on an Abbott Architect c8000 automated clinical chemistry analyzer (Abbott Diagnostics, Abbott Laboratories, Abbott Park, IL, USA). Desialylated (carbohydrate-deficient) transferrin (CDT) was measured on Siemens BN Prospec immunoassay according to the instructions of the manufacturer (Siemens Diagnostics, Erlangen, Germany). The results are expressed as percentages (%) of total transferrin. The concentrations of interleukins (IL-6, IL-8, IL-10), TNF-α and TGF-β were determined using Quantikine high sensitivity ELISA kits (R&D Systems, Abingdon, Science Park, UK). The concentrations of serum CTx and PINP were determined using electro-chemiluminescence immunoassay kits (Roche Diagnostics, Espoo, Finland). Serum PIIINP and ICTP were analyzed by radioimmunological procedures (Orion Diagnostica, Espoo, Finland). Serum HA concentrations were determined using an enzyme-linked binding protein assay from Corgenix (Tejon St. Westminster, CO, USA). All measurements were carried out in SFS-EN ISO 15189:2013 accredited laboratory.

### 4.3. Measurements of IgA Antibody Titers against tTG and Acetaldehyde-Modified Antigens

The IgA antibodies against tissue transglutaminase were measured using Thermo Scientific Phadia Tissue Transglutaminase IgA Antibody Assay (Thermo Fisher Scientific, Waltham, MA, USA) according to the instructions of manufacturer. The results are expressed as units/milliliter (U/mL). The analyses for the IgA antibodies against the acetaldehyde-derived epitopes in proteins (HbAch-IgA) were carried out as previously described [37,58]. In essence, acetaldehyde-modified antigen was prepared in vitro using human hemoglobin as a carrier protein. Erythrocytes were lysed with polyoxyethylene ether, 0.1% *v*/*v* in borate buffer (Hemolysis Reagent, DIAMAT^TM^ Analyzer System, Bio-Rad, Hercules, CA, USA), and incubated for 35 min at +37 °C to remove unstable Schiff bases. The hemolysate was brought into a hemoglobin protein concentration of 12 mg/mL with PBS and stored frozen in aliquots at −70 °C prior to use. Acetaldehyde diluted in PBS was added to aliquots of the freshly prepared hemoglobin, containing 12 mg protein/mL, to obtain a final acetaldehyde concentration of 10 mM, which has been previously shown to effectively lead to the generation of immunogenic epitopes in acetaldehyde-protein condensates [11,37,58]. Protein adducts were reduced by addition of sodium cyanoborohydride (10 mM) and mixing for 5 h at +4 °C to create stable condensates. For measuring the antibody titers, microtiter plates (Nunc-Immuno Plate, Maxisorb^TM^, Thermo Scientific, Waltham MA, USA, Roskilde, Denmark) were coated with acetaldehyde-modified hemoglobin or corresponding unmodified proteins (background) in PBS (3 µg protein in 100 µL/well) [37,58]. Optical densities of color reactions were read at 405 nm with Anthos HTII microplate reader (Anthos Labtec Instruments, Salzburg, Austria).

### 4.4. Statistical Methods

The levels of biomarkers are shown as medians with interquartile ranges (IQR) and analyzed between three subgroups by Kruskal–Wallis analysis of variance followed by Mann–Whitney U test as post hoc test. Mean age among the study subgroups was compared with analysis of variance, and due to unequal variances, the post hoc pairwise comparisons were made using Tamhane’s T2 test. Gender distribution among the subgroups was compared using Pearson’s Chi-square test. The correlations between the study variables were evaluated by Spearman’s rank correlation coefficient (r_s_). The area under the receiver operating characteristic (ROC) curve (AUC) was used to compare the predictive ability of various biomarkers in differentiating between the alcoholic and control groups. A *p*-value of <0.05 was considered statistically significant. Statistical analyses were carried out using IBM SPSS Statistics 28.0 (IBM Corp., Armonk, NY, USA).

## Figures and Tables

**Table 1:** Main demographic characteristics of the study population.

	ALD	AUD without Liver Disease	Healthy Controls			
	N = 83	N = 105	N = 88	*p* ^a^	*p* ^b^	*p* ^c^
Age, mean (SD)	49.7 (11.3)	43.3 (10.6)	48.5 (16.1)	<0.0005	0.921	0.031
Men, *n* (%)	61 (73.5)	90 (85.7)	52 (59.1)	0.036	0.047	<0.0005
Women, *n* (%)	22 (26.5)	15 (14.3)	36 (40.9)			
Ethanol consumption, g/week, mean (SD)	680 (650)	950 (622)	3.0 (8.3)		<0.0005	<0.0005

^a^ ALD vs. AUD without liver disease, ^b^ ALD vs. controls, ^c^ AUD without liver disease vs. controls.

**Table 2:** The discriminative values (AUC) for the different biomarkers in differentiating between ALD or AUD without liver disease from healthy controls. The *p* values refer to differences in AUCs in comparisons of differences between the alcohol-consuming groups.

		AUC (95% CI)		
Biomarker		ALD	AUD without Liver Disease	*p*
tTG-IgA	tissue transglutaminase IgA antibodies	0.95 (0.91–0.99)	0.59 (0.48–0.71)	<0.0005
HbAch-IgA	IgA antibodies against protein adducts with acetaldehyde	0.68 (0.59–0.77)	0.63 (0.54–0.73)	0.472
CDT%	carbohydrate-deficient transferrin	0.87 (0.80–0.94)	0.87 (0.82–0.93)	0.957
GT	gamma glutamyl transferase	0.92 (0.88–0.96)	0.90 (0.85–0.94)	0.356
ALP	alkaline phosphatase	0.93 (0.89–0.97)	0.78 (0.71–0.84)	<0.0005
IL-6	interleukin 6	0.88 (0.83–0.94)	0.71 (0.63–0.80)	0.001
IL-8	interleukin 8	0.88 (0.82–0.94)	0.72 (0.64–0.80)	0.002
TNF-α	tumor necrosis factor-α	0.97 (0.94–1.00)	0.65 (0.56–0.74)	<0.0005
IL-10	interleukin 10	0.36 (0.27–0.45)	0.45 (0.36–0.55)	0.156
TGF-β	transforming growth factor-β	0.26 (0.18–0.34)	0.58 (0.48–0.68)	<0.0005
PIIINP	aminoterminal propeptide of type III procollagen	0.93 (0.89–0.97)	0.38 (0.29–0.48)	<0.0005
PINP	aminoterminal propeptide of type I procollagen	0.71 (0.61–0.80)	0.31 (0.19–0.43)	<0.0005
HA	hyaluronic acid	0.86 (0.80–0.92)	0.45 (0.35–0.55)	<0.0005
ICTP	carboxy-terminal telopeptide of type I procollagen	0.70 (0.57–0.83)	0.62 (0.47–0.77)	0.431
CTx	degradation product of type I collagen (CrossLaps)	0.87 (0.80–0.93)	0.51 (0.38–0.64)	<0.0005

**Table 3:** Correlations between the study parameters. The strengths and directions of relationships between the numerical values are emphasized using color.

	tTG-IgA	HbAch-IgA	CDT%	GT	ALP	IL-6	IL-8	TNF-α	IL-10	TGF-β	PIIINP	PINP	HA	ICTP	CTx
Autoantibodies															
tTG-IgA	1.000														
HbAch-IgA	0.462	1.000													
Markers of alcohol consumption and liver status															
CDT%	0.413	0.156	1.000												
GT	0.487	0.141	0.462	1.000											
ALP	0.466	0.134	0.304	0.599	1.000										
Mediators of inflammation															
IL-6	0.581	0.269	0.400	0.465	0.476	1.000									
IL-8	0.535	0.111	0.374	0.546	0.547	0.490	1.000								
TNF-α	0.591	0.174	0.258	0.433	0.565	0.586	0.567	1.000							
IL-10	−0.051	−0.082	0.098	−0.065	−0.141	0.098	−0.040	−0.019	1.000						
TGF-β	−0.366	−0.102	−0.174	−0.128	−0.244	−0.241	−0.171	−0.221	0.164	1.000					
Markers of fibrogenesis															
PIIINP	0.634	0.211	0.087	0.248	0.442	0.484	0.468	0.595	−0.130	−0.354	1.000				
PINP	0.189	0.126	−0.154	−0.152	0.292	0.137	0.246	0.387	−0.084	−0.180	0.601	1.000			
HA	0.575	0.186	0.087	0.347	0.428	0.494	0.557	0.550	−0.183	−0.473	0.647	0.379	1.000		
ICTP	0.482	0.088	0.232	0.253	0.338	0.317	0.308	0.329	−0.022	−0.218	0.295	0.317	0.240	1.000	
CTx	−0.495	−0.113	−0.106	−0.302	−0.349	−0.117	−0.200	−0.436	0.148	0.310	−0.361	0.113	−0.352	−0.007	1.000

For abbreviations, see Table 2.

## Data Availability

The datasets generated during the current study are not publicly available due to restrictions relating to confidential patient information but are available from the corresponding author on reasonable request.

## Abbreviations

ALD	alcoholic liver disease
ALP	alkaline phosphatase
AUD	alcohol use disorder
CTx, (CrossLaps)	crosslinked isomerized fragment of type I collagen
CDT	desialylated (carbohydrate-deficient) transferrin
ECM	extracellular matrix
GT	gamma-glutamyl transferase
HA	hyaluronic acid
ICTP	carboxy-terminal telopeptide of type I procollagen
IL	interleukin
LPS	lipopolysaccharide
PIIINP	aminoterminal propeptide of type III procollagen
PINP	aminoterminal propeptide of type I procollagen
TNF	tumor necrosis factor
TGF	transforming growth factor
